# Degradation Characteristics of Decommissioned Wind Turbine Blade Composites in Subcritical and Supercritical Fluids

**DOI:** 10.3390/ma19163428

**Published:** 2026-08-13

**Authors:** Yu Ru, Yuzhe Li, Nan Li, Jingchun Huang, Yifan Bao, Yu Qiao

**Affiliations:** 1 School of Energy and Power Engineering, Huazhong University of Science and Technology, Wuhan 430074, China; 2 Beijing Huaneng Yangtze Environmental Technology Research Institute Co., Ltd., Beijing 102209, China; 3 Huaneng Yangtze Environmental Technology Co., Ltd., Beijing 102209, China

**Keywords:** retired wind turbine blades, subcritical/supercritical fluid, glass fiber recycling, chemical recovery

## Abstract

This study investigates the degradation behavior of decommissioned wind turbine blade composites in organic fluid systems, with particular focus on the subcritical acetic acid route. Supercritical acetone and supercritical n-butanol were used as screening media, while retired-blade glass fiber-reinforced polymer (GFRP) composites and laboratory-prepared glass fiber/epoxy composites were used to compare resin removal and fiber recovery behavior. The screening results showed that supercritical acetone and supercritical n-butanol caused partial matrix degradation but left visible organic residues on recovered fibers, whereas subcritical acetic acid produced cleaner fiber surfaces under lower-pressure conditions. The effects of temperature and reaction time were then analyzed in the subcritical acetic acid system. At 280 °C for 60 min, the epoxy resin degradation rate reached 99.81%, and the recovered glass fibers retained 96.49% of their tensile strength. Gas chromatography–mass spectrometry (GC–MS) analysis indicated that the liquid products mainly contained phenols, esters, and other oxygenated organics, with bisphenol A derivatives as representative components. These products suggest a coupled degradation process involving epoxy network swelling, bond cleavage, fragment release, and secondary acetylation in acetic acid. The boiling-point difference between acetic acid and the main degradation products, together with the product distribution obtained after recovered-acid addition, indicates the potential of acetic acid reuse. These findings support subcritical acetic acid as a promising medium for resin removal and glass-fiber recovery from decommissioned wind turbine blade composites.

## 1. Introduction

With the expansion of global wind power capacity, the number of decommissioned wind turbine blades is rapidly increasing. Wind turbine blades are mainly composed of glass fiber-reinforced polymer (GFRP) composites, whose thermosetting resin matrix makes recycling and disposal difficult. Traditional disposal methods such as landfill, open stacking, and incineration cause environmental pressure and fail to realize efficient resource recovery. Therefore, the development of effective recycling technologies for retired wind turbine blade composites has become an important research topic.

Recent studies have explored various recycling routes for fiber-reinforced composites used in wind turbine blades. Rani et al. [1] investigated the recycling of glass fibers from blade composites and pointed out that blade waste is expected to grow rapidly with the expansion of installed wind power capacity. Liu et al. [2] compared the economic performance of several end-of-life disposal strategies. Jani et al. [3] summarized the recycling and reuse technologies for blade materials, including mechanical, thermal, and chemical recovery methods. Majewski et al. [4] emphasized that end-of-life wind turbine blade management should consider landfill restrictions, recycling capacity, environmental regulation, and material recovery pathways from a policy perspective.

Among the existing approaches, chemical recovery has attracted considerable attention because it enables the removal of resin while maintaining relatively high fiber strength. Kooduvalli et al. [5] compared pyrolysis and solvent hydrolysis routes and reported that solvent-based recovery showed advantages in energy consumption and environmental performance. Protsenko et al. [6,7] demonstrated that glass fibers could be chemically recovered from epoxy-based GFRP systems in reactive media, while Ballout et al. [8] proposed a mild solvent hydrolysis method that produced recycled fibers with properties close to those of virgin materials. These studies indicate that suitable chemical environments can promote resin decomposition while preserving the structural integrity of reinforcing fibers.

Thermochemical technologies have also been widely investigated for composite recycling. Protsenko et al. [9] used supercritical ethanol to treat epoxy/glass fiber composites and achieved effective resin removal while maintaining fiber strength. Muzyka et al. [10] reported the simultaneous recovery of resin and glass fibers using a small-molecule solubilization system. Shen et al. [11] reviewed recycling strategies for fiber-reinforced polymers in wind turbine blades from the perspective of green chemistry, emphasizing the importance of balancing environmental impact and material performance during chemical degradation.

Although considerable progress has been made, several limitations remain. Many studies have focused on simplified composite plates or single material systems, whereas real wind turbine blades contain complex multilayer structures, including glass fiber/epoxy laminates, structural adhesives, and polyurethane materials. In addition, systematic investigations of degradation behavior, fiber properties, and liquid-phase product composition under different subcritical and supercritical organic fluid conditions remain limited.

In this work, glass fiber-reinforced epoxy resin composites obtained from decommissioned wind turbine blades were selected as the main feedstock, and laboratory-prepared GFRP plates were used as controlled reference samples for product identification and mechanism discussion [12]. The novelty of this study lies in combining real retired-blade GFRP treatment, organic-fluid screening, recovered-fiber property evaluation, gas chromatography–mass spectrometry (GC–MS) product analysis, and recovered-acid participation within the same experimental framework. Supercritical acetone, supercritical n-butanol, and subcritical acetic acid were first compared to clarify their different effects on resin removal and fiber recovery. After subcritical acetic acid showed more favorable degradation behavior under lower-pressure conditions, the subsequent analysis focused on temperature and time effects, liquid-product evolution, possible acetolysis and secondary acetylation pathways, and the practical potential of acetic acid reuse. This design links solvent selection with degradation chemistry and recovered-fiber preservation, providing experimental evidence for chemical treatment of decommissioned wind turbine blade composites.

## 2. Materials and Methods

### 2.1. Composite Materials of Retired Wind Turbine Blades and Sub-/Supercritical Fluid System

**Figure 1:**
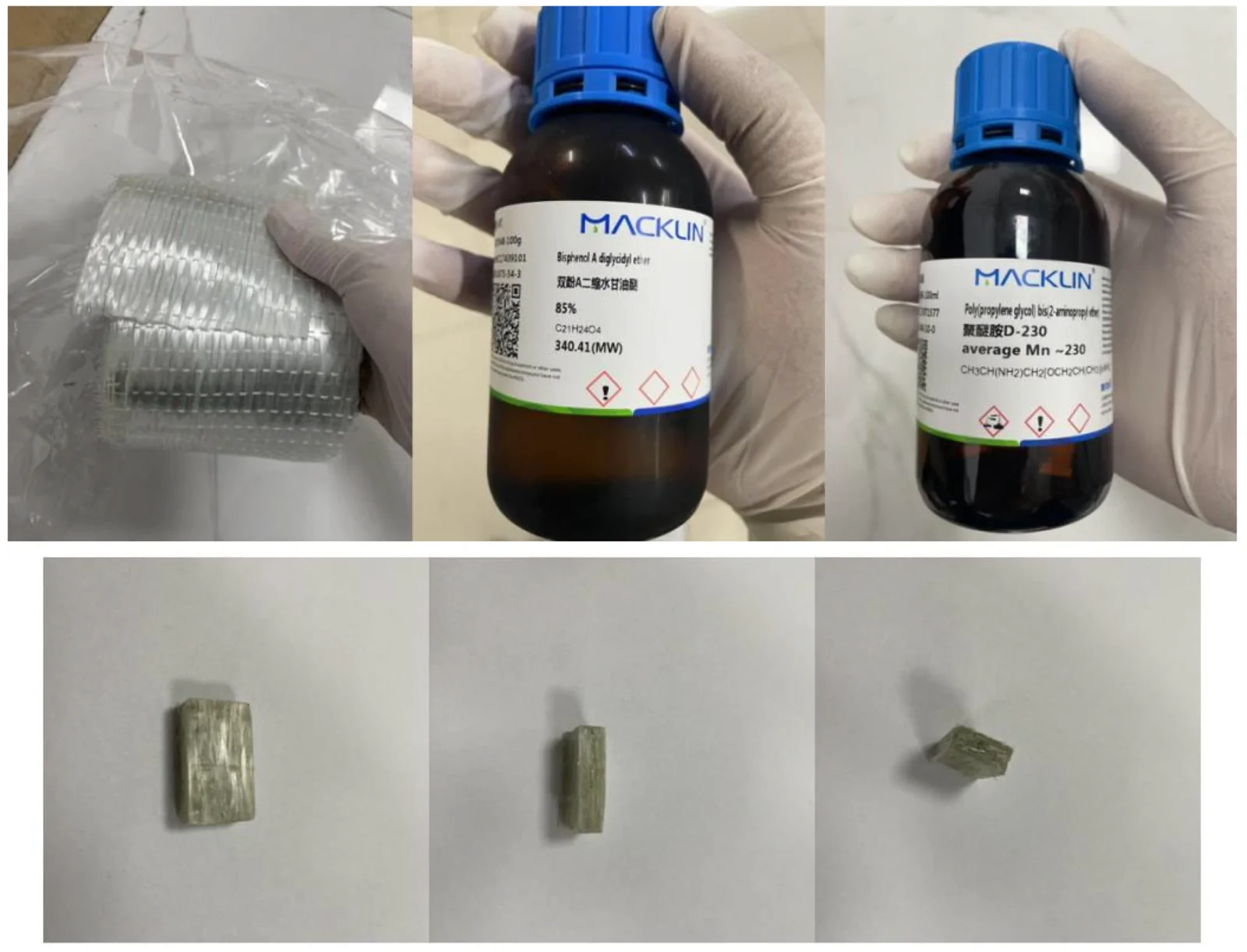
Alkali-free E-glass fiber cloth, bisphenol A diglycidyl ether, polyether amine D-230, and laboratory-prepared glass fiber-reinforced polymer (GFRP) plates. The Chinese text on the reagent-bottle labels identifies bisphenol A diglycidyl ether and polyether amine D-230, respectively.

Composite samples from retired wind turbine blades were obtained from a full-scale blade that had completed its service cycle. The blade root and blade body were mechanically dismantled on site, and the glass fiber-reinforced epoxy resin laminate (GFRP) sections were then cut into regular sheets in the laboratory for use as degradation samples in subcritical and supercritical fluid experiments [13]. To support mechanistic analysis and improve the controllability of material composition, laboratory-prepared GFRP plates were also introduced as reference samples. The laboratory-prepared GFRP was fabricated using alkali-free E-glass woven roving (EWR600, 600 g/m^2^; Jushi Group Co., Ltd., Tongxiang, China) as the reinforcement and a matrix system composed of bisphenol A diglycidyl ether (BADGE, 85%) and polyether amine D-230, both supplied by Shanghai Macklin Biochemical Co., Ltd. (Shanghai, China), followed by vacuum impregnation and curing. The raw materials and the prepared plates are shown in Figure 1. Analytical-grade acetone, n-butanol, and glacial acetic acid from Sinopharm Chemical Reagent Co., Ltd. (Shanghai, China) were selected as the working media in the fluid-system screening stage. Supercritical acetone and supercritical n-butanol were used only to evaluate whether supercritical organic solvents could effectively promote resin removal from retired blade GFRP, whereas subcritical acetic acid was chosen as the main reaction medium for the subsequent in-depth analysis. Before degradation, both retired blade samples and laboratory-prepared GFRP samples were dried at 105 °C to constant weight, and their initial masses were recorded using an electronic balance. All samples were then coded uniformly to distinguish material source and to support subsequent mass-balance calculation and degradation analysis.

### 2.2. Test Device and Physical Property Characterization Method

**Figure 2:**
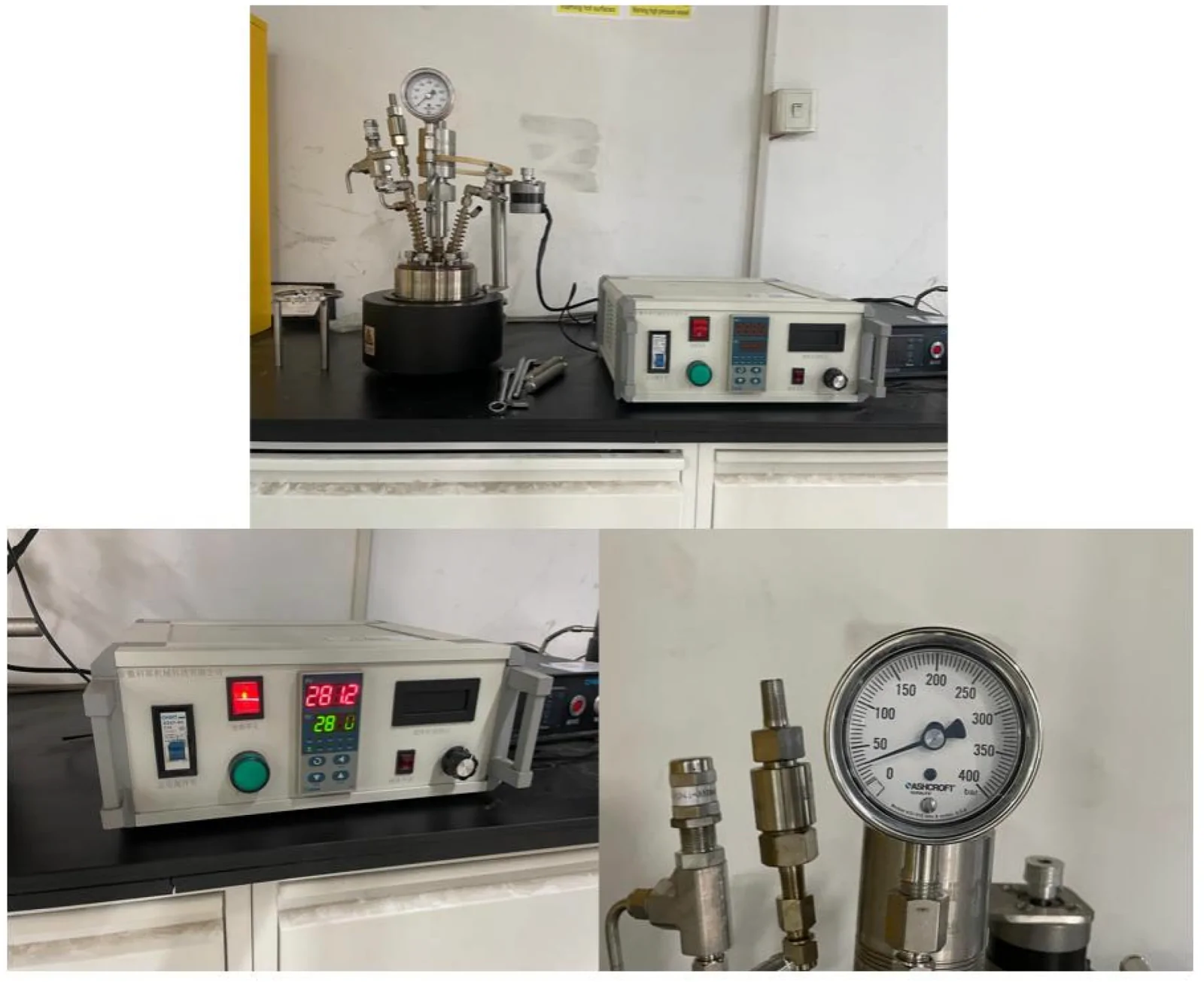
Physical arrangement of the high-pressure degradation reactor system.

The degradation experiments of retired wind turbine blade composites in subcritical and supercritical fluids were carried out using a custom-built 50 L reactor (Weihai Zhongda Machinery Co., Ltd., Weihai, China). The apparatus consisted of a stainless-steel autoclave, an electric heating furnace, a temperature-control and pressure-display unit, a safety relief valve, and inlet/outlet pipelines. The system allowed batch degradation tests to be performed in acetone, n-butanol, and acetic acid under controlled temperature and pressure conditions [14]. Before each experiment, dried GFRP specimens with uniform dimensions and a fixed volume of organic fluid were added into the reactor, so that the solvent/sample ratio remained constant for all comparable experiments. After the sealing state had been checked, nitrogen was introduced twice to replace the air in the vessel and maintain an inert atmosphere. The reactor was then heated to the target temperature at the preset heating rate and held for the required reaction time. During the reaction, temperature was monitored using a K-type thermocouple, and pressure was read from an Ashcroft analog gauge (Ashcroft Inc., Stratford, CT, USA) mounted on the reactor cover. After completion of the reaction, the autoclave was removed from the furnace and cooled in a metal container filled with cold water. When the temperature approached room temperature, the pressure was released slowly and the reactor was opened to separate the liquid and solid products. The physical arrangement of the high-pressure degradation system is shown in Figure 2. The recovered glass fiber was first rinsed repeatedly with deionized water to remove acetic acid or other soluble residues, then immersed in acetone and treated in a KQ-500DE ultrasonic cleaner (40 kHz, 500 W; Kunshan Ultrasonic Instruments Co., Ltd., Kunshan, China) to dissolve organic matter adsorbed on the fiber surface. After a second rinsing with deionized water, the fiber was dried at 105 °C to constant weight. All mass measurements were carried out using a BSA224S-CW balance (220 g/0.1 mg; Sartorius, Göttingen, Germany) [15]. Liquid-phase products were analyzed by GC–MS using an Agilent 8860 gas chromatograph coupled with a 5977B mass selective detector (Agilent Technologies, Santa Clara, CA, USA). Before testing, the liquid samples were diluted with acetone at a volume ratio of 1:1, and the solvent delay was set to 3.5 min to avoid interference from the major peaks of acetone and acetic acid. The relative contents of the low-molecular-weight compounds were determined by peak-area normalization. The tensile properties of the recovered glass fibers were measured using an Instron 5943 system with a 100 N load cell and single-filament grips (Instron, Norwood, MA, USA). Bluehill Universal (version not recorded) controlled the tests. Only valid monofilament data with a clear fracture position in the gauge region were retained for strength analysis. Each condition was tested in triplicate. Reactor control used its built-in PLC/touchscreen program (no separate version). During the degradation process, the reactor temperature was monitored in real time by a K-type thermocouple and recorded by a temperature controller, while the internal pressure was read from the gauge mounted on the reactor cover.

### 2.3. Subcritical/Supercritical Fluid Degradation Process and Operating Condition Setting

The degradation experiments were carried out in a batch high-pressure reactor using GFRP samples obtained from decommissioned wind turbine blades. In the fluid-system screening stage, the samples were separately treated in supercritical acetone, supercritical n-butanol, and subcritical acetic acid. The acetone system was examined at 260–300 °C and 52–75 bar, the n-butanol system at about 360 °C and 75 bar, and the acetic acid system at 200–280 °C and 22–30 bar. These conditions correspond to supercritical acetone and supercritical n-butanol because their critical parameters are approximately 235 °C/47 bar and 290 °C/44 bar, respectively. In contrast, acetic acid remained in the subcritical region under the present conditions because its critical point is approximately 318 °C/57 bar. After screening identified subcritical acetic acid as the preferred reaction medium, further experiments in this system were designed by varying temperature and holding time to analyze resin removal, recovered-fiber strength retention, and liquid-phase product evolution [16]. In each run, a weighed composite sample and a fixed amount of solvent were loaded into the reactor, the vessel was purged with nitrogen, heated to the target temperature, and then held for the specified time. After reaction, the reactor was cooled to room temperature, the pressure was released slowly, and the liquid and solid products were collected for subsequent analysis. Identical sample size, heating program, and post-treatment procedures were used to ensure comparability among different fluid systems and among the subcritical acetic acid conditions. The resin degradation rate was calculated from the removed resin mass relative to the initial resin mass in the GFRP sample. The initial resin mass fraction was obtained from burn-off analysis of the untreated laminate, and the dried residual solid was weighed after degradation, washing, and drying. The degradation rate was calculated using Equation (1):

(1) $$Dr=\frac{m0wr-mr}{m0wr}\times 100\%$$

where $D_{r}$ is the resin degradation rate, $m_{0}$ is the initial mass of the dried GFRP sample, $w_{r}$ is the initial resin mass fraction, and $m_{r}$ is the residual resin mass remaining in the cleaned solid residue. The residual resin mass was calculated from the difference between the dried residual solid mass and the initial glass-fiber mass. The mass balance was checked using the initial composite mass, recovered solid residue, washing loss, and liquid-phase products.

### 2.4. Cleaning of Recovered Fibers and Tensile Strength Characterization

After degradation, the recovered fibers were separated from the residual solid and rinsed repeatedly with deionized water until the washing liquid became clear, in order to remove acetic acid and soluble low-molecular-weight products. The fibers were then immersed in acetone and treated in the KQ-500DE ultrasonic cleaner described above to dissolve residual organic matter attached to the surface. After a second deionized-water rinse, the fibers were dried at 105 °C to constant weight. Changes in fiber appearance and mass during cleaning and drying were recorded to assist the evaluation of resin-removal effectiveness [17].

The tensile properties of the recovered glass fibers were measured using the Instron 5943 system. The dried fibers were mounted on paper frames and tested at a constant tensile rate and gauge length. Only single filaments that fractured within the valid gauge region were included in the strength calculation, while multi-strand specimens and invalid fracture data were excluded. The strength retention rate was calculated by comparing the tensile strength of recovered fibers with the baseline glass fibers obtained from untreated GFRP after matrix removal. Triplicate experiments referred to independent degradation runs, and tensile-strength data were collected from valid filaments recovered from these runs. All quantitative results are presented as mean values with standard deviations. For monofilament tensile testing, 32–40 valid single filaments were retained for each degradation condition. Weibull analysis was used to obtain the characteristic tensile strength and Weibull modulus of the recovered fibers. One-way analysis of variance (ANOVA) was applied to evaluate the effects of temperature and reaction time on degradation rate and fiber-strength retention, with *p* < 0.05 considered statistically significant [18]. No separate statistical software was recorded.

## 3. Results

### 3.1. Screening of Different Fluid Systems for Degradation of Retired Blade GFRP

**Figure 3:**
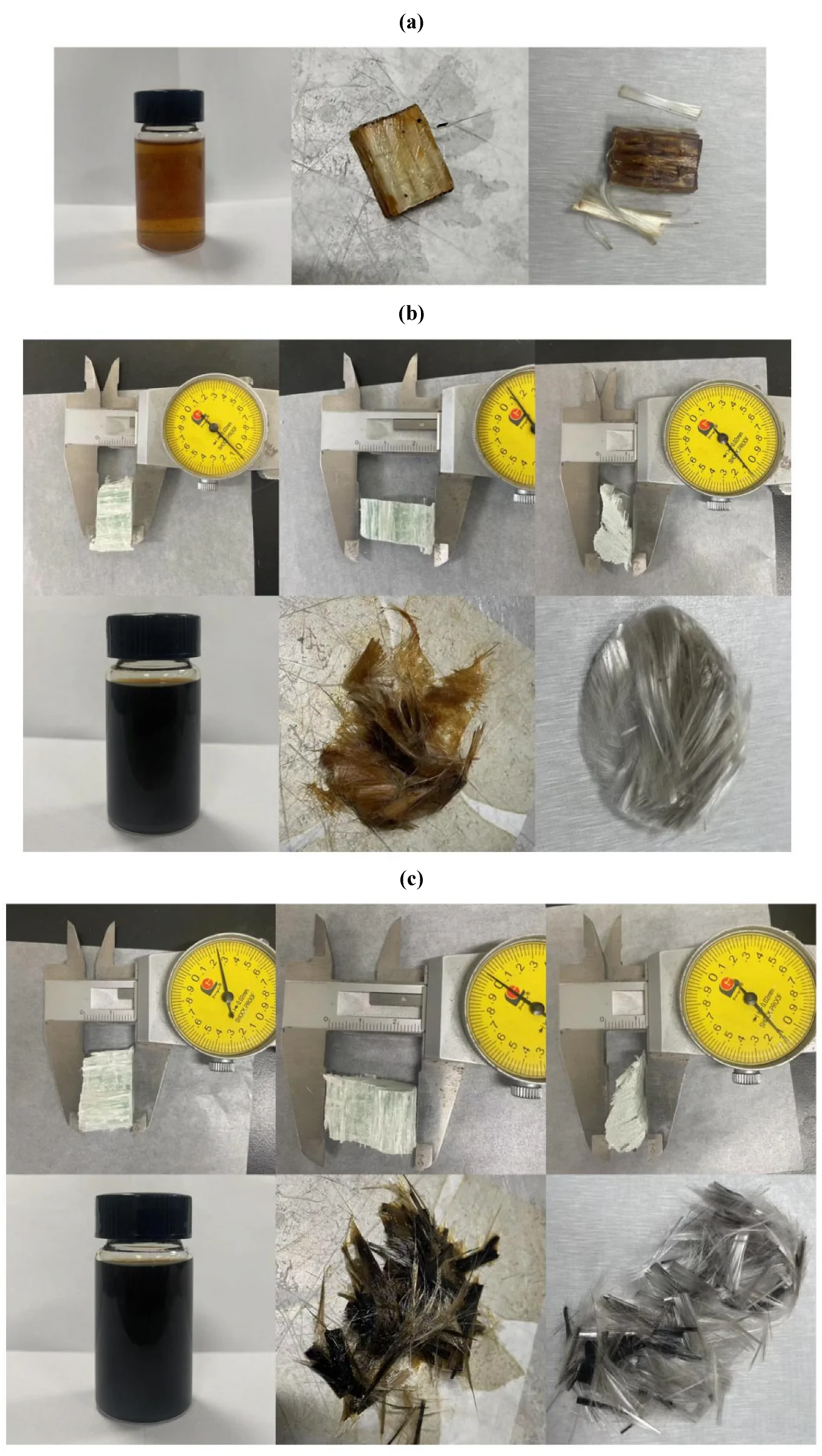
Degradation morphology of retired blade GFRP treated in supercritical organic solvents: (**a**) supercritical acetone at 260 °C; (**b**) supercritical acetone at 300 °C; (**c**) supercritical n-butanol at 360 °C.

As shown in Figure 3, composites of retired wind turbine blades under different sub/supercritical organic fluid conditions show significantly different degradation behaviors. As shown in Figure 3a, the liquid phase obtained after treatment in supercritical acetone at 260 °C remained light yellow and relatively transparent, suggesting that only a limited amount of resin-derived products was transferred into the solvent phase. The recovered composite still preserved an integral plate-like shape, and only slight surface discoloration and local edge peeling could be observed. No obvious delamination, fiber loosening, or bulk collapse appeared in the specimen [19]. These visual features indicate that under this condition, supercritical acetone mainly induced mild swelling of the epoxy matrix rather than effective cleavage of the cross-linked network, which is consistent with the very low overall degradation degree observed in the mass-loss result.

Figure 3b presents the degradation morphology of the sample treated in supercritical acetone at 300 °C for 1 h. Compared with Figure 3a, the liquid phase became markedly darker, indicating that more organic fragments were released into the solvent. The recovered solid no longer showed a smooth compact surface; instead, brown resin-derived residues were clearly attached to the exposed fiber region [20]. After water washing and acetone cleaning, part of the deposited material was removed, and the underlying fiber bundle became visible. However, the recovered fibers still remained in a relatively dense and compact bundle with low fluffiness, indicating that although the higher temperature promoted resin cracking, the degradation products still adhered strongly to the fiber surface and limited complete resin removal.

As shown in Figure 3c, treatment in supercritical n-butanol at 360 °C resulted in a much darker reaction liquid and more severe surface carbonized residues than those observed in the acetone system. The recovered solid was broken into several sheet-like fiber regions, indicating that the resin matrix had undergone substantial softening and fragmentation during the reaction. Nevertheless, each separated region still retained the original fabric-like arrangement of the glass fibers, which means that the reinforcement framework was not completely dispersed [21]. After post-cleaning, a considerable portion of the fibers turned gray rather than white, and black residues were still visible in some local regions. This morphology suggests that the n-butanol system could trigger resin decomposition, but the generated products showed strong adhesion and were more difficult to remove completely from the fiber surface.

**Figure 4:**
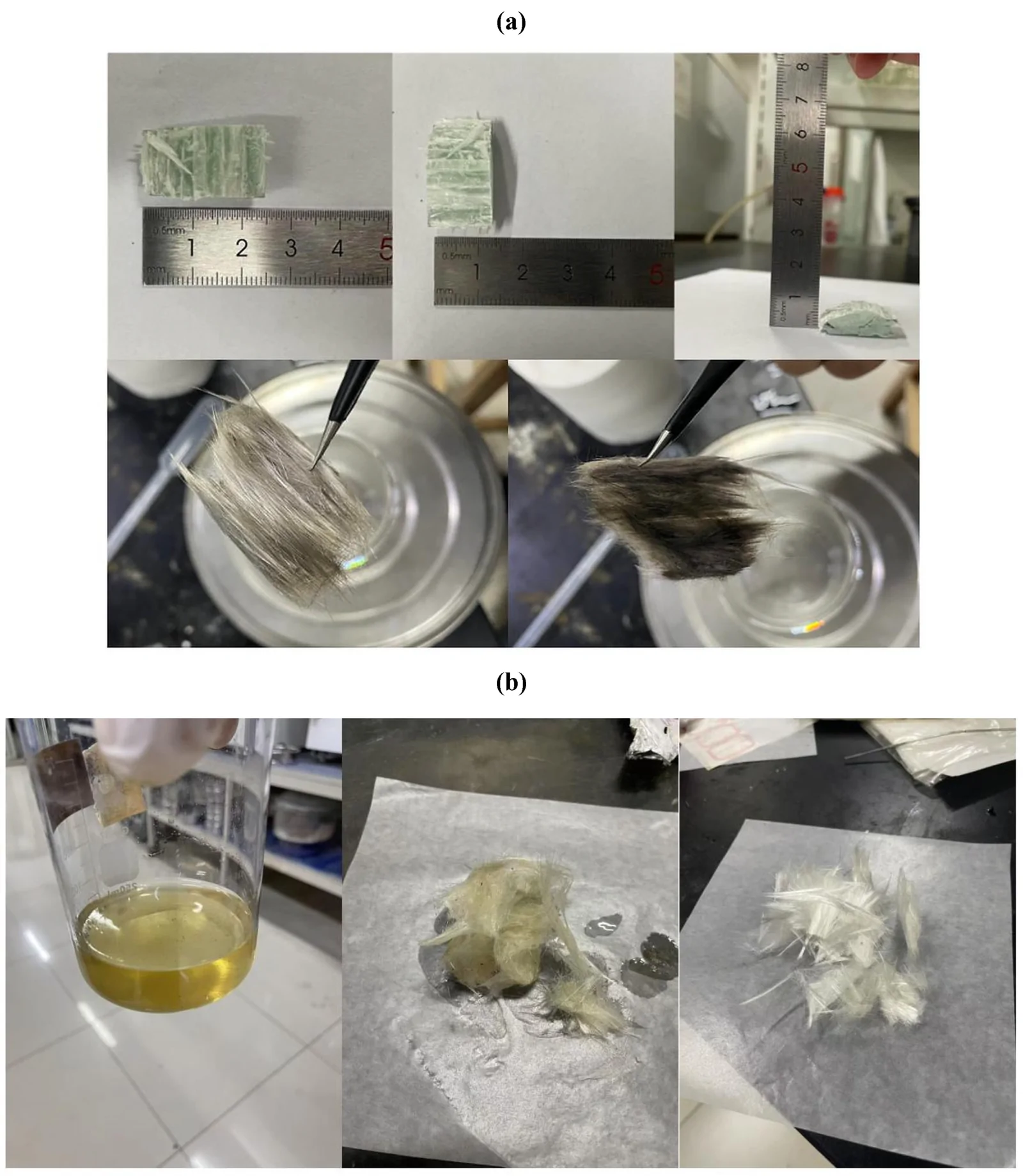
Degradation morphology of retired blade GFRP treated in subcritical acetic acid at 260 °C: (**a**) reaction for 1 h; (**b**) reaction for 3 h.

Figure 4 illustrates the morphological changes during the degradation of the composite under the subcritical acetic acid system. Figure 4a shows the morphology of the composite treated in subcritical acetic acid at 260 °C for 1 h. The specimen maintained its macroscopic plate geometry, but the side-view image indicates that the internal structure had become looser and partially separated along the laminate direction. After reaction, the recovered fiber bundle remained continuous as a whole, rather than collapsing into small fragments [22]. A clear difference could be observed between the two sides of the recovered material: one side appeared relatively clean, with the fiber alignment distinctly exposed, whereas the other side still carried dark residual deposits. This feature indicates that subcritical acetic acid had already penetrated into the resin-rich region and promoted evident matrix decomposition within 1 h, although local residual resin was still present in part of the laminate.

As shown in Figure 4b, when the treatment time in subcritical acetic acid was prolonged to 3 h under the same temperature condition, the liquid phase remained fluid and the solid residue changed more significantly. The recovered material no longer preserved a compact plate morphology, but was converted into loose fiber bundles mixed with softened organic residues immediately after reaction [23]. During subsequent washing, brown substances were continuously released from the surface, indicating that soluble degradation products and loosely attached oligomers were still being removed. After acetone extraction and drying, the final recovered fibers became obviously whiter, more dispersed, and fluffier than those obtained after 1 h treatment. This morphological change confirms that prolonging the reaction time in the subcritical acetic acid system can further enhance resin removal and improve fiber cleanliness, which is in agreement with the higher weight-loss ratio observed under this condition.

The above comparison shows that supercritical acetone and supercritical n-butanol served as screening media for evaluating the degradation behavior of retired blade GFRP in different organic fluid systems [24]. Under the tested conditions, both supercritical solvent systems produced visible resin-derived residues on the recovered fibers, while subcritical acetic acid showed more favorable resin-removal behavior and cleaner fiber morphology under lower-pressure conditions. Therefore, the subsequent discussion focuses on the subcritical acetic acid system. The screening comparison was therefore used to identify the most suitable medium for subsequent in-depth analysis, while the quantitative optimization of resin degradation and fiber-strength retention was conducted in the selected subcritical acetic acid system.

The screening results summarized in Table 1 and Table 2 show clear differences among the three organic fluid systems. Supercritical acetone at 260 °C caused only limited resin removal, with a degradation rate of 21.34%, while increasing the temperature to 300 °C improved the degradation rate to 56.72% but still left obvious organic residues on the recovered fibers. Supercritical n-butanol achieved a higher degradation rate of 78.15%, whereas its recovered fibers showed lower strength retention because of high-temperature exposure and strongly adhered dark residues. In contrast, subcritical acetic acid reached 97.02% degradation at 260 °C and 99.81% at 280 °C, while maintaining high recovered-fiber strength retention. Based on these results, subcritical acetic acid was selected as the main reaction medium for subsequent analysis of temperature, reaction time, liquid-phase products, degradation pathway, and recovered-acid participation [25].

The following sections therefore examine how temperature and reaction time affect resin degradation and fiber retention in subcritical acetic acid, and then analyze liquid-phase product evolution, degradation pathways, and solvent-recycling potential.

### 3.2. Effects of Temperature and Time on Resin Removal and Fiber Retention in the Subcritical Acetic Acid System

**Figure 5:**
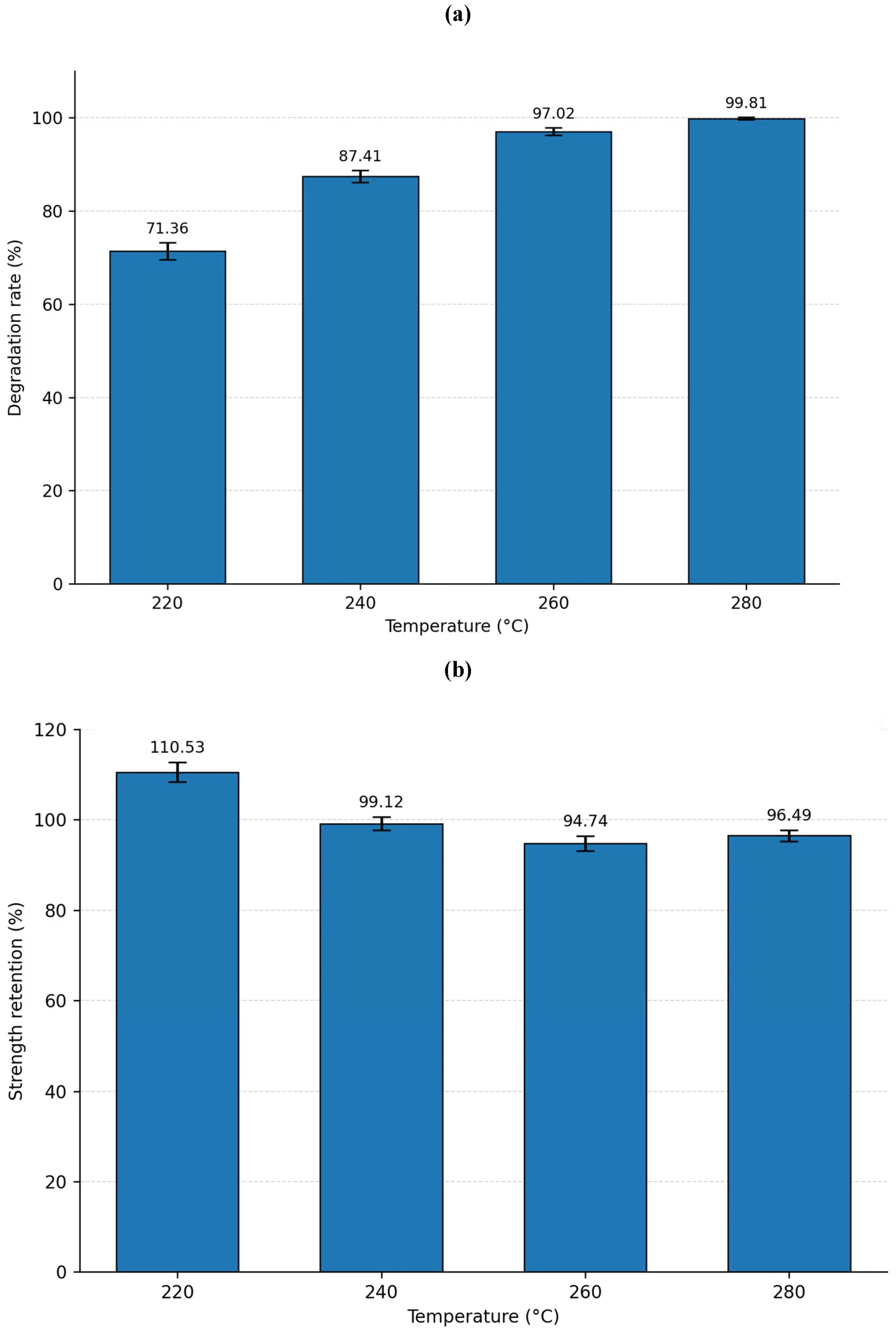
Temperature dependence of (**a**) the GFRP degradation rate and (**b**) the strength retention of recovered glass fibers under subcritical acetic acid conditions. Error bars represent standard deviations.

In the subcritical acetic acid system, the degradation of GFRP from retired wind turbine blades shows that the epoxy matrix gradually breaks and transfers to the liquid phase, while the mechanical properties of the recovered glass fibers vary with reaction temperature [26]. The degradation rate at different temperatures is shown in Figure 5a, and the corresponding strength retention rate of recovered glass fiber is shown in Figure 5b. When the temperature increased from 220 to 280 °C, the resin degradation rate increased from 71.36% to 99.81%. At 220 °C, the epoxy network was only partially cleaved, and residual organic matrix still adhered to the glass-fiber surface. When the temperature increased to 240 and 260 °C, the degradation rate reached 87.41% and 97.02%, respectively, indicating that subcritical acetic acid promoted swelling and bond cleavage of the epoxy network more effectively at elevated temperature. At 280 °C, the degradation rate reached 99.81%, suggesting that most of the resin matrix had been converted into soluble products. The apparent strength retention reached 110.53% at 220 °C, which may be related to residual epoxy coating on the fiber surface. Such residual coating could share part of the tensile load and lead to an apparent value higher than that of the baseline fiber. As temperature increased, resin removal exposed the original surface defects of the glass fibers, and the strength retention values changed to 99.12%, 94.74%, and 96.49%, respectively. The slight increase from 260 to 280 °C may reflect scatter in single-filament tensile testing rather than a true strengthening effect. Overall, resin degradation was nearly complete between 260 and 280 °C, while the recovered fibers still retained high mechanical stability.

**Figure 6:**
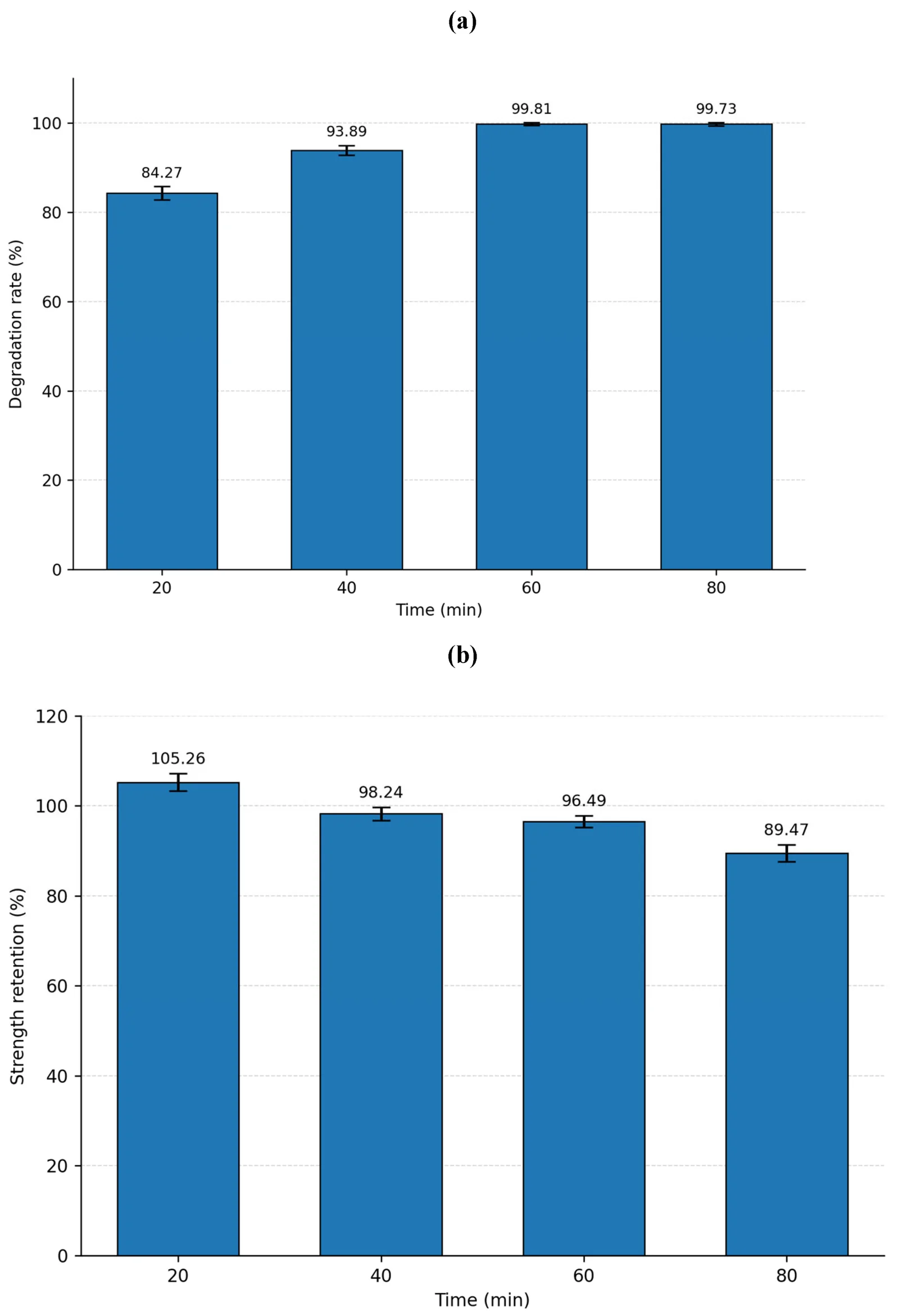
Effect of degradation time on (**a**) the GFRP degradation rate and (**b**) the strength retention of recovered glass fibers under subcritical acetic acid conditions at 280 °C. Error bars represent standard deviations.

Under the fixed temperature of 280 °C, the degradation behavior of the composite material was significantly affected by reaction time [27]. The degradation rate of GFRP at different degradation times is shown in Figure 6a, and the corresponding strength retention rate of recovered glass fiber is shown in Figure 6b. At 280 °C, reaction time strongly affected resin removal and recovered-fiber strength. The degradation rate increased from 84.27% at 20 min to 93.89% at 40 min and further to 99.81% at 60 min, showing that the epoxy network underwent progressive swelling, bond cleavage, and dissolution into the liquid phase. Extending the reaction time to 80 min produced only a slight change in degradation rate, with a value of 99.73%, indicating that resin removal had nearly reached equilibrium. In contrast, the strength retention rate decreased from 105.26% at 20 min to 98.24% at 40 min and 96.49% at 60 min, then dropped sharply to 89.47% at 80 min. The higher apparent value at 20 min was probably associated with residual resin on the fiber surface, while the marked decrease at 80 min suggests that prolonged exposure to high-temperature acidic media damaged the glass-fiber surface. Therefore, 280 °C for 60 min provided a better balance between resin removal and fiber-strength retention.

The tensile results in Table 3 show that the number of valid single filaments for each condition ranged from 32 to 40, which provided a more reliable statistical basis than triplicate degradation runs alone. The apparent strength retention above 100% at 220 °C and 20 min was associated with residual resin coating on the fiber surface, which could share part of the tensile load and affect the apparent fracture behavior. ANOVA results indicated that temperature had a significant effect on degradation rate (*p* < 0.001) and strength retention (*p* < 0.05), while reaction time also significantly affected both degradation rate and recovered-fiber strength (*p* < 0.001). Weibull analysis further confirmed that the recovered fibers treated at 280 °C for 60 min retained stable tensile behavior, whereas prolonged treatment for 80 min reduced the characteristic strength.

Scanning electron microscopy (SEM) and surface-composition results in Table 4 support the visual observations of recovered-fiber cleanliness. Fibers recovered from supercritical acetone and n-butanol still showed continuous organic coatings, resin islands, or carbonaceous residues, which were consistent with their higher residual carbon contents. In contrast, the fibers recovered from subcritical acetic acid at 280 °C for 60 min displayed a cleaner surface and a residual carbon content close to the baseline fiber. When the reaction time was extended to 80 min, the residual organic content remained low, but slight surface etching appeared, which explains the decrease in tensile strength retention under prolonged acidic treatment.

**Figure 7:**
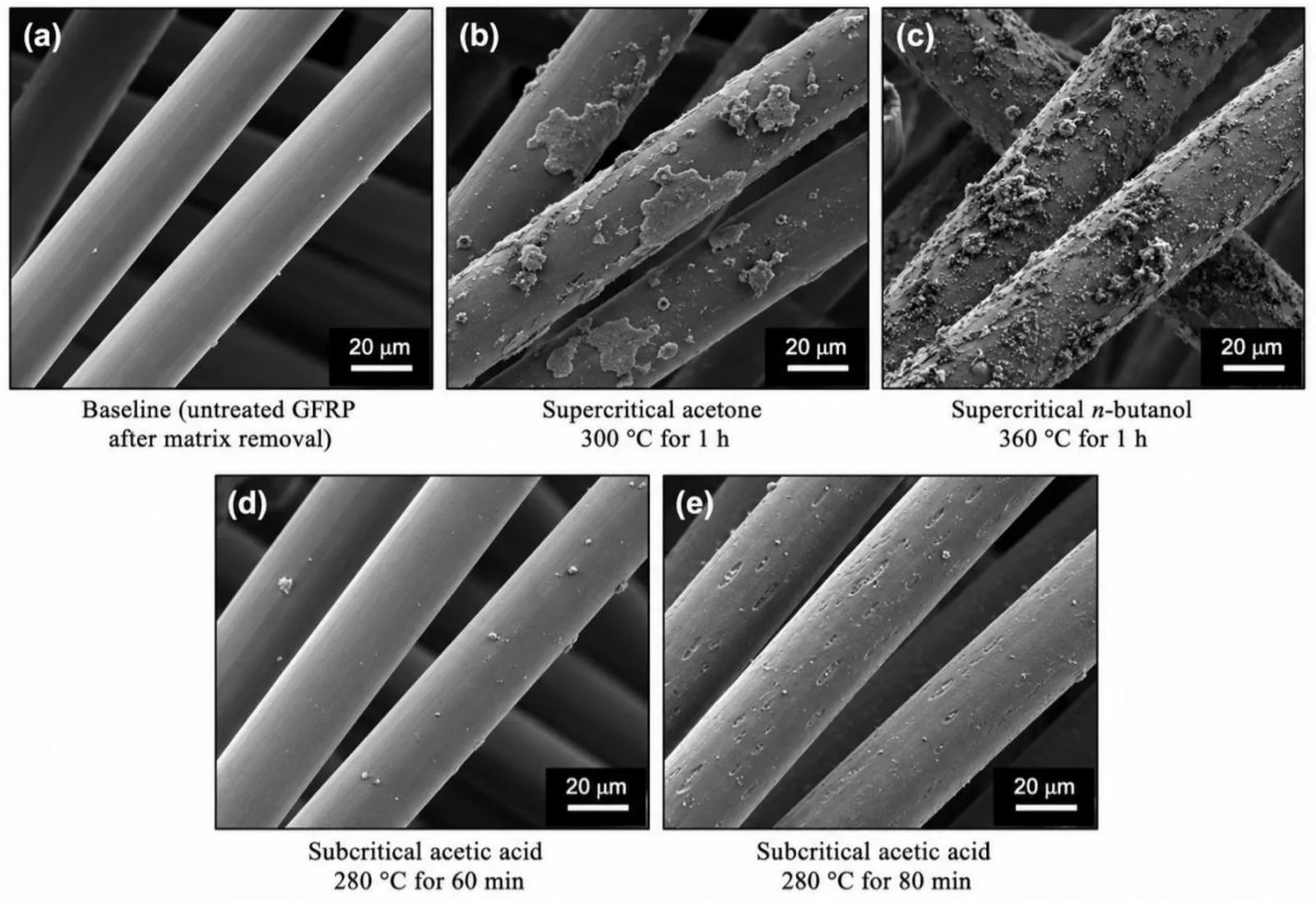
SEM images of recovered glass fibers after different degradation treatments: (**a**) baseline glass fiber obtained from untreated GFRP after matrix removal; (**b**) supercritical acetone at 300 °C for 1 h; (**c**) supercritical n-butanol at 360 °C for 1 h; (**d**) subcritical acetic acid at 280 °C for 60 min; (**e**) subcritical acetic acid at 280 °C for 80 min.

To further verify the surface cleanliness and possible damage of the recovered glass fibers, representative fibers obtained under different degradation conditions were examined by SEM. The selected samples included the baseline glass fiber obtained from untreated GFRP after matrix removal, fibers recovered from supercritical acetone and supercritical n-butanol treatments, and fibers recovered from subcritical acetic acid under the optimized and prolonged reaction conditions. As shown in Figure 7, this comparison was used to distinguish residual organic attachment from fiber surface etching and to support the discussion of strength retention.

### 3.3. GC–MS Analysis and Degradation Pathway in the Subcritical Acetic Acid System

To clarify the chemical transformation of the epoxy matrix in the subcritical acetic acid system, the liquid-phase products from reference epoxy structural adhesive were analyzed by GC–MS as an epoxy-rich model system. The results were used to assist product assignment and mechanism discussion for the GFRP degradation system. The liquid phase consisted mainly of the reaction medium acetic acid and a series of soluble organic compounds generated from epoxy-resin depolymerization. According to the GC–MS results, the major tentatively identified products included phenol, phenyl acetate, bisphenol A diacetate, and several oxygen-containing aromatic or ester derivatives. The assignments were mainly based on library matching, and the relative contents were calculated by peak-area normalization without response-factor correction. Therefore, the GC–MS results were used as semi-quantitative evidence for product-distribution analysis rather than as absolute concentration data. These compounds indicate that the cross-linked epoxy network was gradually converted into low-molecular-weight products in the acidic high-temperature environment.

From the viewpoint of molecular structure, the cured epoxy resin in GFRP is a highly cross-linked network mainly composed of bisphenol A-type epoxy units and amine-curing segments, in which ether bonds (C–O–C), C–N bonds, and aromatic structural units coexist. Under subcritical acetic acid conditions, acetic acid acts not only as a solvent but also as a reactive acidic medium. At the initial stage of degradation, acetic acid penetrates into the resin network and induces swelling of the cross-linked structure, thereby increasing the distance between polymer chains and facilitating the diffusion of reactive molecules into the matrix interior. With increasing temperature, the relatively labile ether bonds and C–N bonds are preferentially cleaved, leading to the release of aromatic and oxygen-containing intermediates. Some of these intermediates remain as phenolic compounds, whereas others undergo further esterification or acetylation in the acetic acid environment to form more stable products. Therefore, the degradation process in subcritical acetic acid can be understood as a coupled process involving network swelling, acid-assisted bond cleavage, fragment release, and secondary acetylation, rather than simple physical dissolution of the resin. Compared with supercritical acetone and n-butanol, acetic acid provides both solvent penetration and an acidic reactive environment. The acidic medium facilitates cleavage of labile ether and C–N bonds in the epoxy network, while hydroxyl- or phenolic intermediates generated during depolymerization can further react with acetic acid to form acetate derivatives. This acetolysis-dominated pathway explains why cleaner fibers and bisphenol A-derived ester products were observed in the subcritical acetic acid system.

**Figure 8:**
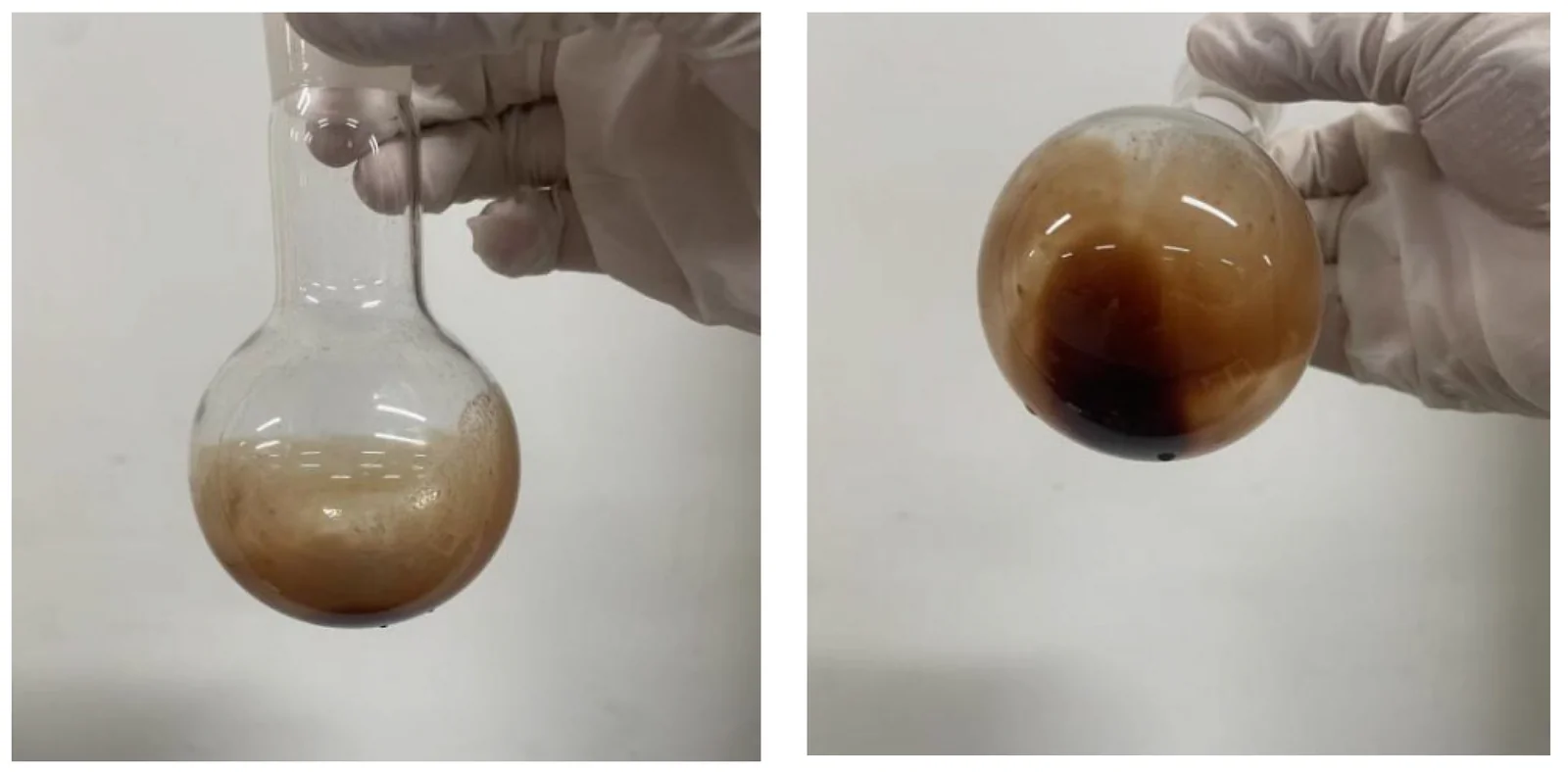
Macroscopic morphology of liquid-phase products obtained from GFRP degradation in subcritical acetic acid.

Under the reaction condition of 280 °C for 60 min, a large amount of brown liquid-phase products was generated, indicating that most of the resin had already been converted into soluble or semi-soluble organic species. As shown in Figure 8, the liquid product exhibits a distinct macroscopic stratified appearance, with a lighter yellow-brown upper liquid phase and a darker condensed fraction accumulated in the lower region. No obvious large undegraded resin blocks can be observed, suggesting that the epoxy matrix was not merely softened or detached from the fiber surface, but was chemically depolymerized into low-molecular-weight products. The darker lower-phase accumulation is considered to be associated with heavier aromatic oxygen-containing fragments or partially condensed products formed during cooling, whereas the upper liquid phase mainly contains acetic acid and dissolved low-molecular-weight degradation compounds. This macroscopic morphology is consistent with the proposed degradation mechanism and further confirms the conversion of the original insoluble thermoset matrix into liquid-phase products in the subcritical acetic acid system.

To further evaluate the stability and separation characteristics of the main liquid-phase components, the boiling points of representative products were summarized, as listed in Table 5. Acetic acid has a much lower boiling point than the major phenolic and ester products generated from epoxy degradation, whereas compounds such as bisphenol A diacetate and diethylene glycol dipropionate remain in a relatively high-boiling range. This distribution indicates that once the epoxy network is cleaved and converted into aromatic and oxygen-containing derivatives, the solvent and degradation products can theoretically be separated by reduced-pressure evaporation and distillation. Therefore, the subcritical acetic acid system not only promotes resin depolymerization, but also provides a basis for evaluating post-reaction solvent recovery.

To further verify the chemical origin of the liquid-phase products, the degradation solution of epoxy structural adhesive treated in subcritical acetic acid at 280 °C was analyzed by GC–MS. As shown in Table 6, the liquid products from retired blade GFRP were dominated by phenolic compounds, aromatic acetate derivatives, oxygenated esters, and bisphenol A diacetate-related products. The identified products accounted for 66.31% of the total chromatographic peak area, while 33.69% corresponded to minor, overlapping, or low-confidence peaks. Compared with the epoxy structural adhesive reference, the GFRP system showed similar major product categories but slightly lower relative abundance of bisphenol A diacetate-related products. This difference can be attributed to the restricted contact between acetic acid and the resin matrix inside the glass-fiber framework, as well as the more complex composition of retired blade GFRP.

To directly identify the liquid-phase products generated from the actual retired blade GFRP system, the degradation solution obtained under the optimized subcritical acetic acid condition was further analyzed by GC–MS. The condition of 280 °C for 60 min was selected because it provided a high resin degradation rate while maintaining favorable recovered-fiber strength retention. Different from the epoxy structural adhesive reference, the retired blade GFRP sample contained a glass-fiber framework and more complex blade-derived components; therefore, its chromatogram was presented separately to clarify the product distribution of the real composite system.

**Figure 9:**
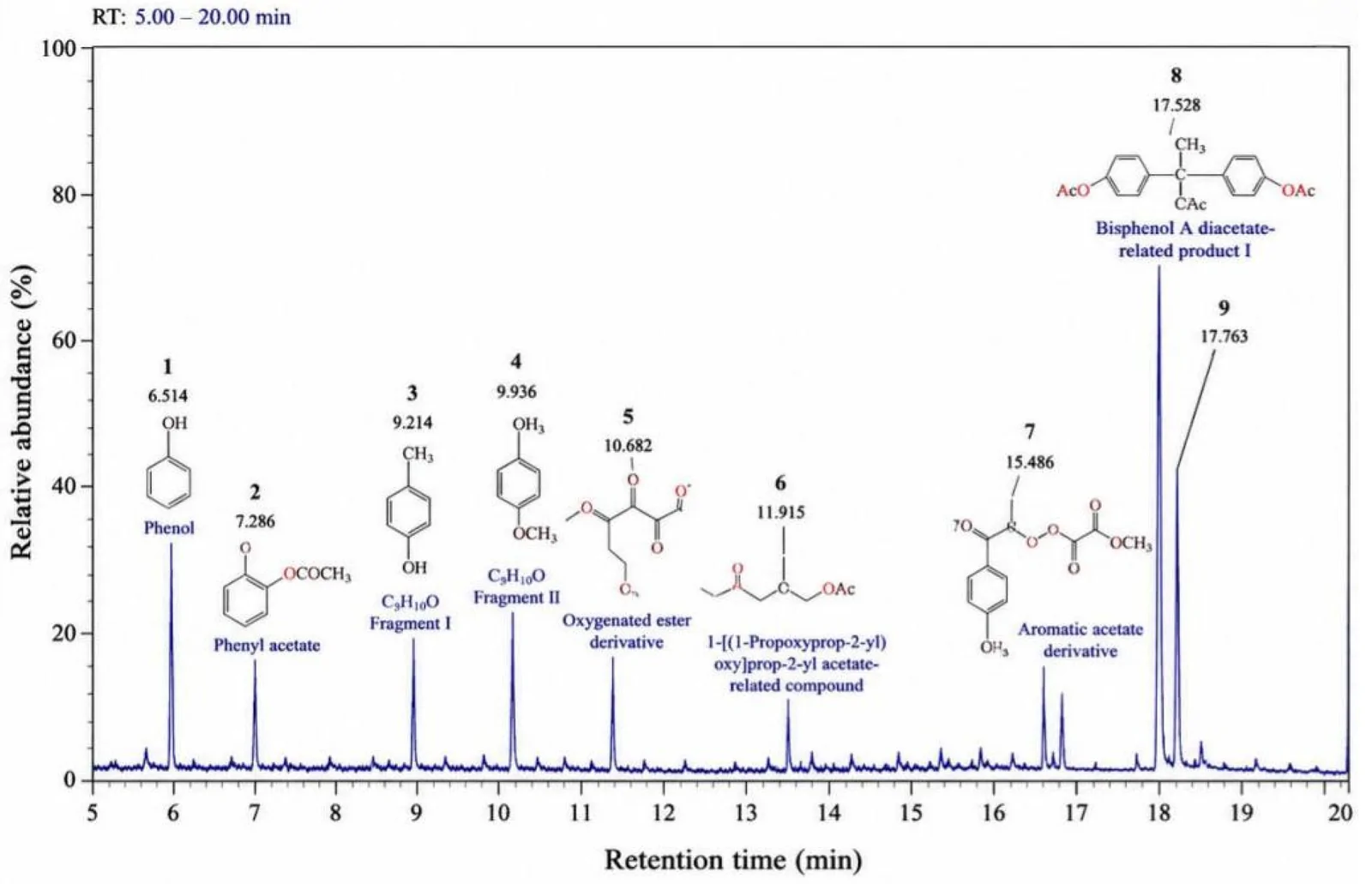
Gas chromatography–mass spectrometry (GC–MS) chromatogram of liquid-phase products from retired blade GFRP degraded in subcritical acetic acid at 280 °C for 60 min. Peak numbers correspond to the compounds listed in Table 6.

As shown in Figure 9, several obvious peaks appeared in the retention-time range of approximately 6–18 min, indicating that the cross-linked epoxy matrix in retired blade GFRP was converted into a series of soluble organic products during subcritical acetic acid treatment. The major peaks were concentrated in the middle and late retention-time regions, suggesting the formation of phenolic compounds, aromatic acetate derivatives, oxygenated esters, and bisphenol A-derived products. These results confirm that the real GFRP system followed a degradation pathway similar to that of the epoxy-rich reference system, while the glass-fiber framework mainly affected product release and relative product distribution.

**Figure 10:**
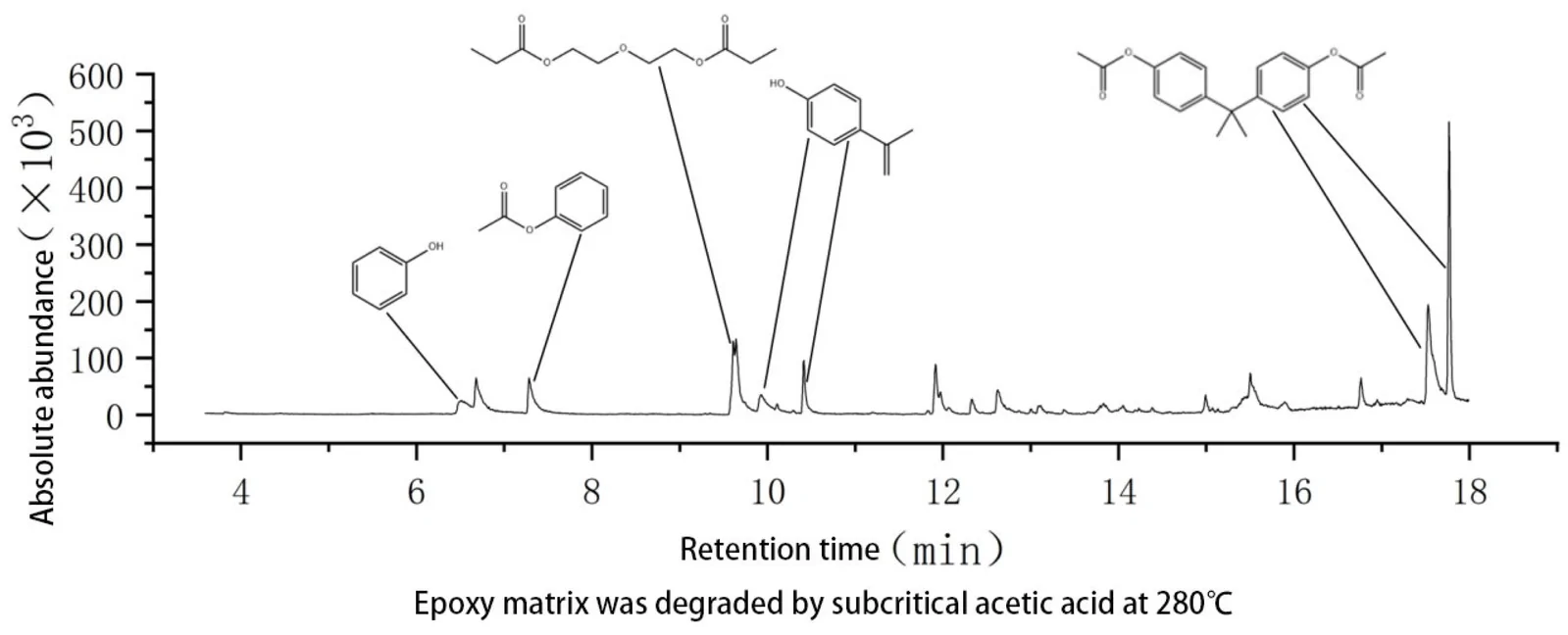
Chromatogram of liquid-phase products from epoxy structural adhesive degraded by subcritical acetic acid at 280 °C.

As shown in Figure 10, several obvious chromatographic peaks appeared in the retention-time range of 6–18 min, indicating that a variety of organic products with different structures were generated in the reaction system. The peaks were mainly concentrated in the middle retention-time region, suggesting that the products were dominated by medium-molecular-weight aromatic compounds and their oxygen-containing derivatives.

By combining chromatographic peak positions with mass-spectrometric identification, a series of typical degradation products were assigned, including phenol, phenyl acetate, diethylene glycol dipropionate, 1-[(1-propoxyprop-2-yl)oxy]prop-2-yl acetate, 1-acetoxy-3-allyl-4-(1,3-diacetoxypropoxy)benzene, and bisphenol A diacetate. Among these compounds, bisphenol A diacetate-related products showed the highest relative abundance, with two major peaks at 17.526 and 17.766 min. These two adjacent peaks may correspond to closely related bisphenol A-derived acetate structures or chromatographically separated isomeric/derivative species rather than two independent identical compounds. Therefore, they are discussed here as bisphenol A diacetate-related products, and their exact structural assignment requires further confirmation with authentic standards. This result indicates that the subcritical acetic acid environment not only cleaved the epoxy resin network, but also promoted the acetylation of bisphenol A-derived intermediates to form relatively stable ester derivatives. In contrast, the lower contents of phenol and phenyl acetate reflect the gradual release and secondary transformation of aromatic fragments during degradation.

The compounds listed in Table 7 accounted for 71.18% of the total chromatographic peak area. The remaining 28.82% corresponded to minor peaks, overlapping peaks, or low-abundance components that could not be assigned with sufficient confidence by library matching. Therefore, the product distribution in Table 7 was used to identify the dominant degradation products and infer the main reaction pathway, rather than to provide a complete quantitative composition of the liquid phase.

Comparison between the GFRP system and the pure epoxy structural adhesive system shows that the major product categories are generally consistent. Except for a small amount of 3-(diethylamino)-1,2-propanediol detected in the GFRP system, the main products showed only limited differences in species and relative abundance. This result indicates that glass fiber does not fundamentally alter the chemical degradation pathway of the epoxy resin, but mainly affects the reaction at the physical level by limiting the contact area between the resin matrix and acetic acid within the composite structure. As a result, the subcritical acetic acid system can be regarded as a chemically effective route for epoxy depolymerization, while the presence of the glass-fiber framework mainly influences degradation rate and product release behavior.

### 3.4. Product Distribution Under Recovered Acetic Acid Participation and Solvent Recycling Potential

**Figure 11:**
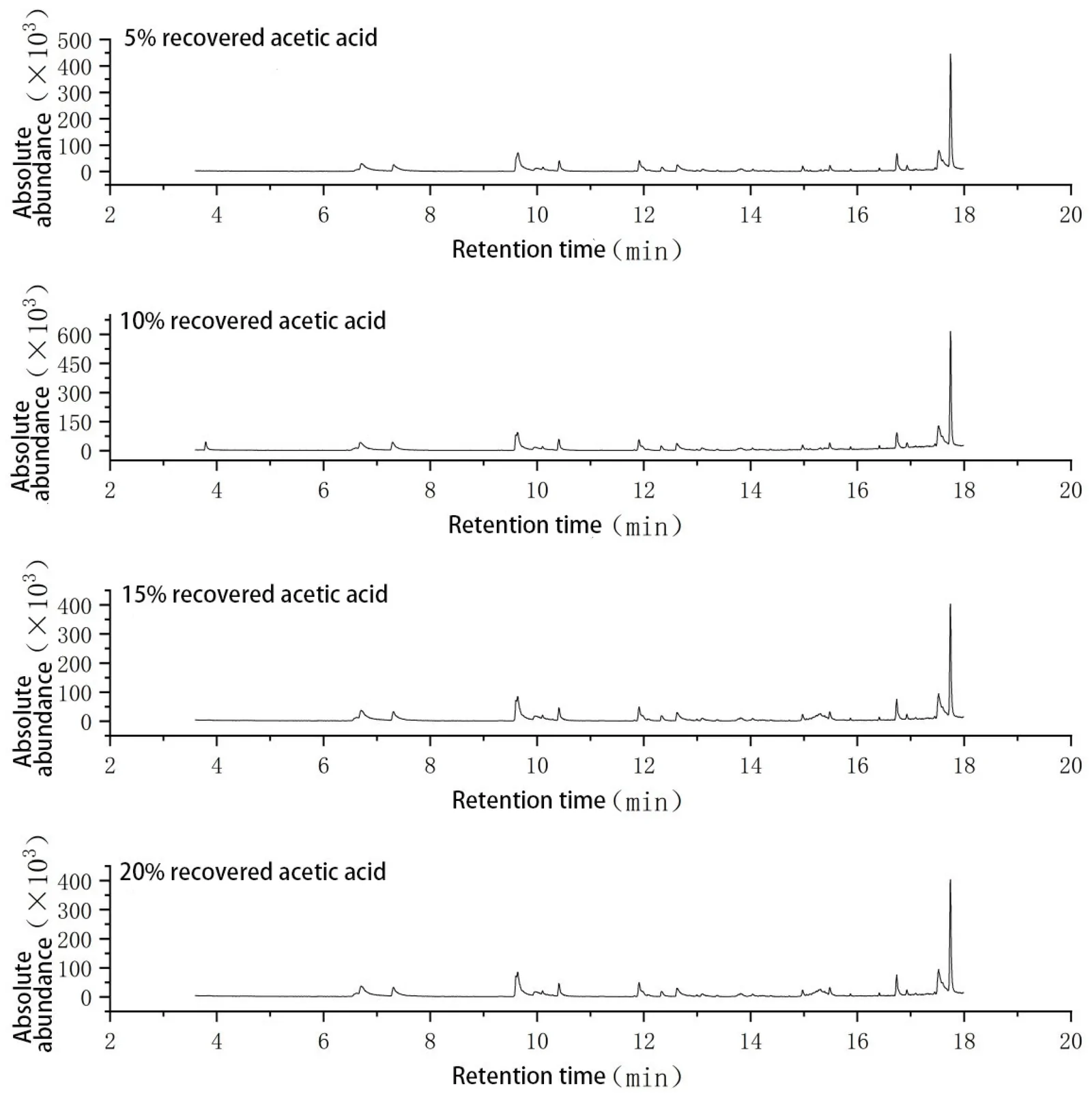
Chromatograms of liquid products obtained by degrading GFRP with different addition ratios of recovered acetic acid.

In order to evaluate the applicability of recycled acetic acid in the subcritical degradation system, 5, 10, 15, and 20 wt.% recovered acetic acid were added to the reaction system in the GFRP degradation experiment, and the composition changes in liquid phase products under different addition ratios were analyzed. The liquid phase products obtained after the reaction at 280 °C for 1 h showed dark brown flow state, and no obvious gelatinous clumps or undegraded solid fragments were observed in the system, which indicated that the epoxy matrix could achieve sufficient degradation reaction in the subcritical acetic acid environment. The results of GC analysis of the liquid phase products obtained under different proportions of recovered acetic acid are shown in Figure 11.

It can be observed from the chromatogram that multiple obvious peaks appeared in the liquid phase products under each experimental condition, indicating that a variety of low-molecular-weight organic compounds were formed during degradation in subcritical acetic acid. On the whole, the main peak positions of different systems were basically consistent, indicating that the addition of recycled acetic acid did not change the main path of the degradation reaction of epoxy resin. The chromatographic peaks were mainly distributed in the middle retention time region, and the high-intensity peak appearing around 17~18 min was the most significant characteristic peak in the system, corresponding to bisphenol A derivatives, which were one of the main products in the degradation system. With the change in the proportion of recovered acetic acid, the chromatogram of each group showed some differences in peak intensity and peak number, indicating that the addition of recovered acetic acid would affect the distribution of degradation products.

As shown in Table 8, the main product category changed with the proportion of recovered acetic acid, but the dominant chromatographic regions remained similar. This result indicates that reintroduced recovered acid affected product distribution but did not fundamentally change the degradation pathway. The higher content of bisphenol A diacetate-related products at 5 wt.% recovered acid compared with the structural-adhesive reference may be related to differences in feedstock composition, resin accessibility, and secondary acetylation during degradation.

The product distribution shown in Table 8 indicates that bisphenol A diacetate-related products remained the dominant components at low recovered-acid addition ratios. Their relative content reached 41.67% at 5 wt.% recovered acetic acid and decreased to 31.10% at 10 wt.%, suggesting that bisphenol A-derived fragments still underwent evident acetylation in the acetic acid medium. When the recovered-acid ratio increased to 15 wt.%, phenolic compounds became more prominent, with a relative content of 11.51%, indicating further fragmentation of aromatic intermediates. At 20 wt.%, diethylene glycol dipropionate-related oxygenated esters increased to 14.17%, suggesting that oxygenated ester products were more enriched under higher recovered-acid addition. Overall, recovered-acid addition changed the relative product distribution but did not fundamentally alter the main degradation pathway.

The above chromatographic results show that the main product category changed with the proportion of recovered acetic acid, but the dominant retention-time regions remained similar. This result indicates that reintroduced recovered acid affected the relative distribution of degradation products but did not fundamentally change the main degradation pathway. Under 5 wt.% recovered acetic acid addition, bisphenol A diacetate-related products reached 41.67%, suggesting that bisphenol A-derived fragments were effectively released and acetylated in the acetic acid medium. When the recovered-acid ratio increased to 10 wt.%, the relative content of these products decreased to 31.10%, but they still remained the dominant components. At 15 wt.%, phenolic compounds increased to 11.51%, indicating further fragmentation of aromatic intermediates. At 20 wt.%, diethylene glycol dipropionate-related oxygenated esters increased to 14.17%, showing that higher recovered-acid addition changed the relative distribution of oxygenated products.

The spent liquid phase obtained after GFRP degradation was subjected to reduced-pressure distillation to recover acetic acid. The recovered fraction was analyzed by GC to determine acetic acid purity and then reused in the next degradation cycle under the same condition of 280 °C for 60 min.

The cyclic reuse results in Table 9 show that acetic acid could be recovered from the spent liquid phase with a recovery yield of 86.7% in the first cycle. The recovery yield gradually decreased to 82.6% after the third reuse, while the recovered acetic acid purity remained above 96.0%. The degradation rate decreased slightly from 99.81% with fresh acetic acid to 97.82% after the third reuse, and the recovered-fiber strength retention remained higher than 94%. These results indicate that recovered acetic acid retained most of its degradation ability during short-cycle reuse. Therefore, the solvent-reuse potential of the subcritical acetic acid route is supported not only by boiling-point differences and product-distribution analysis, but also by preliminary cyclic recovery data.

Taken together, the above results indicate that the subcritical acetic acid route retains its basic degradation pathway when recovered acetic acid is reintroduced into the system, although the relative distribution of liquid-phase products changes with the recovered-acid ratio. The cyclic reuse results further show that recovered acetic acid maintained high purity and degradation ability over three reuse cycles. Combined with the temperature- and time-dependent results and the GC–MS-based pathway analysis, these findings suggest that subcritical acetic acid is a promising reaction medium for retired wind turbine blade GFRP treatment and has practical solvent-reuse potential.

## 4. Conclusions

This study screened organic fluid systems for the degradation of retired wind turbine blade GFRP and then focused on the subcritical acetic acid route. Supercritical acetone and supercritical n-butanol caused partial matrix degradation but left visible organic residues on recovered fibers, whereas subcritical acetic acid produced cleaner recovered fibers under lower-pressure conditions. Temperature- and time-dependent experiments indicated that 280 °C for 60 min achieved a favorable balance between resin removal and glass-fiber strength retention. GC–MS analysis showed that the liquid-phase products were mainly phenolic, ester, and oxygen-containing aromatic compounds, with bisphenol A-derived acetate products as representative species. These results support a degradation pathway involving network swelling, acid-assisted bond cleavage, fragment release, and secondary acetylation in acetic acid. The boiling-point difference between acetic acid and major degradation products, together with recovered-acid addition and cyclic reuse experiments, supports the solvent-reuse potential of the subcritical acetic acid route. In the preliminary reuse test, recovered acetic acid maintained a purity above 96.0% after three cycles, while the GFRP degradation rate remained 97.82% and the recovered-fiber strength retention remained 94.36%. The present work provides experimental support for using subcritical acetic acid to remove resin, recover glass fibers, and promote solvent reuse in the treatment of decommissioned wind turbine blade composites. Further work should quantify closed-loop acetic-acid recovery, verify product assignments with standards, and evaluate recovered-fiber surface chemistry and long-term reuse performance.

## Figures and Tables

**Table 1:** Screening comparison of retired blade GFRP degradation in different organic fluid systems.

Fluid System	Reaction Condition	Pressure	Resin-Removal Behavior	Recovered-Fiber Morphology	Screening Evaluation
Supercritical acetone	260 °C, 1 h	52–75 bar	Only slight resin swelling and limited matrix removal were observed.	The specimen still retained an integral plate-like structure, with slight surface discoloration and local edge peeling.	Insufficient resin degradation under this condition.
Supercritical acetone	300 °C, 1 h	52–75 bar	More resin fragments were released into the liquid phase, but residual organics remained evident.	Brown resin-derived residues were attached to exposed fiber regions, and the fiber bundle remained compact after cleaning.	Partial degradation occurred, but fiber cleanliness was limited.
Supercritical n-butanol	360 °C, 1 h	75 bar	Resin softening and fragmentation were more obvious than in acetone.	The recovered solid broke into sheet-like fiber regions, but gray or black residues remained after cleaning.	Resin decomposition was promoted, but residue adhesion was severe.
Subcritical acetic acid	260 °C, 1 h	22–30 bar	The laminate became loose along the layer direction, indicating evident matrix decomposition.	One side of the recovered fiber bundle became relatively clean, with exposed fiber alignment, while local dark residues remained.	More favorable resin-removal behavior under lower pressure.
Subcritical acetic acid	260 °C, 3 h	22–30 bar	Prolonged treatment further promoted resin removal and release of soluble degradation products.	The recovered fibers became whiter, more dispersed, and fluffier after washing, extraction, and drying.	Selected as the preferred medium for subsequent temperature- and time-dependent analysis.

**Table 2:** Quantitative comparison of GFRP degradation performance under representative fluid-screening conditions.

Fluid System	Condition	Pressure	Degradation Rate (%)	Recovered-Fiber Yield (%)	Strength Retention (%)	Main Limitation
Supercritical acetone	260 °C, 1 h	52–75 bar	21.34 ± 1.27	93.86 ± 2.10	101.72 ± 4.38	Resin swelling occurred, but matrix cleavage was limited.
Supercritical acetone	300 °C, 1 h	52–75 bar	56.72 ± 2.64	86.45 ± 2.33	92.36 ± 3.21	Residual resin remained on the recovered fibers.
Supercritical n-butanol	360 °C, 1 h	75 bar	78.15 ± 3.08	80.73 ± 2.84	83.58 ± 3.76	Carbonized or strongly adhered residues remained.
Subcritical acetic acid	260 °C, 1 h	22–30 bar	97.02 ± 0.86	94.18 ± 1.62	94.74 ± 2.12	Local residues remained in resin-rich regions.
Subcritical acetic acid	280 °C, 60 min	22–30 bar	99.81 ± 0.18	92.60 ± 1.75	96.49 ± 2.08	Prolonged reaction may reduce fiber strength.

**Table 3:** Single-filament tensile statistics of recovered glass fibers under subcritical acetic acid conditions.

Variable	Condition	Valid Filaments (n)	Mean Tensile Strength (GPa)	Strength Retention (%)	Weibull Characteristic Strength (GPa)	Weibull Modulus
Baseline	Untreated GFRP after matrix removal	40	1.96 ± 0.08	100.00	2.04	6.42
Temperature	220 °C, 60 min	36	2.17 ± 0.10	110.53	2.25	5.18
Temperature	240 °C, 60 min	38	1.94 ± 0.07	99.12	2.01	6.05
Temperature	260 °C, 60 min	35	1.86 ± 0.06	94.74	1.94	6.76
Temperature	280 °C, 60 min	37	1.89 ± 0.05	96.49	1.97	7.12
Time	280 °C, 20 min	34	2.06 ± 0.09	105.26	2.14	5.63
Time	280 °C, 40 min	36	1.93 ± 0.06	98.24	2.00	6.44
Time	280 °C, 60 min	37	1.89 ± 0.05	96.49	1.97	7.12
Time	280 °C, 80 min	33	1.75 ± 0.07	89.47	1.82	6.38

**Table 4:** Surface characterization of recovered glass fibers under representative conditions. EDS, energy-dispersive X-ray spectroscopy; FTIR, Fourier-transform infrared spectroscopy.

Condition	Fiber Diameter (μm)	SEM Surface Feature	Residual Carbon Content by EDS (%)	FTIR Surface Signal
Baseline fiber	13.4 ± 0.5	Smooth surface with limited attached residue	5.8 ± 0.6	Weak organic signal
Supercritical acetone, 300 °C, 1 h	13.6 ± 0.7	Continuous organic coating and local resin islands	18.7 ± 1.4	Aromatic C=C and C–O–C signals remained
Supercritical n-butanol, 360 °C, 1 h	13.1 ± 0.8	Rough surface with dark particulate residues	22.4 ± 1.8	Strong carbonaceous residue signal
Subcritical acetic acid, 280 °C, 60 min	13.3 ± 0.5	Clean fiber surface with only scattered particles	7.2 ± 0.7	Organic residue signal was weak
Subcritical acetic acid, 280 °C, 80 min	12.8 ± 0.6	Slight surface etching and shallow pits	6.5 ± 0.8	Weak organic signal with more exposed glass surface

**Table 5:** Boiling-point characteristics of representative liquid-phase products obtained from GFRP degradation in subcritical acetic acid.

Compound	Pressure	Boiling Point
Acetic acid	Atmospheric	117.9 °C
Bisphenol A diacetate	0.40 kPa	215 °C
Diethylene glycol dipropionate	Atmospheric	280.3 °C
Phenyl acetate	Atmospheric	196 °C
3-(Diethylamino)-1,2-propanediol	Atmospheric	223 °C
Phenol	Atmospheric	181.1 °C

**Table 6:** Main liquid-phase products from retired blade GFRP degraded in subcritical acetic acid at 280 °C for 60 min.

No.	Retention Time (min)	Molecular Formula	Tentative Compound Assignment	Relative Content (%)
1	6.514	C_6_H_6_O	Phenol	4.62
2	7.286	C_8_H_8_O_2_	Phenyl acetate	5.35
3	9.214	C_9_H_10_O	C9H10O aromatic oxygenated fragment I	4.88
4	9.936	C_9_H_10_O	C9H10O aromatic oxygenated fragment II	5.41
5	10.682	C_10_H_18_O_5_	Oxygenated ester derivative	6.72
6	11.915	C_11_H_22_O_4_	1-[(1-Propoxyprop-2-yl)oxy]prop-2-yl acetate-related compound	3.85
7	15.486	C_18_H_22_O_7_	Aromatic acetate derivative	6.24
8	17.528	C_19_H_20_O_4_	Bisphenol A diacetate-related product I	14.21
9	17.763	C_19_H_20_O_4_	Bisphenol A diacetate-related product II	15.03
			Identified fraction	66.31
			Unidentified or unresolved fraction	33.69

**Table 7:** Main liquid-phase products of epoxy structural adhesive degraded by subcritical acetic acid at 280 °C.

No.	Retention Time/Min	Molecular Formula	Compound	Relative Content
1	6.511	C_6_H_6_O	Phenol	3.80%
2	7.284	C_8_H_8_O_2_	Phenyl acetate	4.91%
3	9.641	C_10_H_18_O_5_	Diethylene glycol dipropionate	8.56%
4	9.927	C_9_H_10_O	Unidentified C9H10O aromatic oxygenated fragment I	3.29%
5	10.414	C_9_H_10_O	Unidentified C9H10O aromatic oxygenated fragment II	4.22%
6	11.827	C_11_H_22_O_4_	1-[(1-Propoxyprop-2-yl)oxy]prop-2-yl acetate	0.23%
7	11.913	C_11_H_22_O_4_	1-[(1-Propoxyprop-2-yl)oxy]prop-2-yl acetate	4.00%
8	13.83	C_11_H_22_O_4_	1-[(1-Propoxyprop-2-yl)oxy]prop-2-yl acetate	1.61%
9	15.5	C_18_H_22_O_7_	1-Acetoxy-3-allyl-4-(1,3-diacetoxypropoxy)benzene	7.11%
10	17.526	C_19_H_20_O_4_	Bisphenol A diacetate	16.68%
11	17.766	C_19_H_20_O_4_	Bisphenol A diacetate	16.77%

**Table 8:** Representative liquid-phase products under different recovered-acid addition ratios.

Recovered Acetic Acid Ratio	Main Enriched Product or Product Group	Relative Content	Interpretation
5 wt.%	Bisphenol A diacetate-related products	41.67%	Enrichment of bisphenol A-derived acetate products
10 wt.%	Bisphenol A diacetate-related products	31.10%	Main degradation pathway remained dominated by bisphenol A fragment acetylation
15 wt.%	Phenolic compounds	11.51%	Further fragmentation of aromatic intermediates became more evident
20 wt.%	Diethylene glycol dipropionate-related oxygenated esters	14.17%	Oxygenated ester products increased under higher recovered-acid addition

**Table 9:** Reuse performance of recovered acetic acid in cyclic GFRP degradation experiments.

Cycle	Acetic Acid Recovery Yield (%)	Recovered Acetic Acid Purity (%)	Degradation Rate (%)	Recovered-Fiber Strength Retention (%)
Fresh acetic acid	—	99.5	99.81 ± 0.18	96.49 ± 2.08
1st reuse	86.7 ± 1.5	97.8 ± 0.6	99.12 ± 0.26	95.93 ± 2.16
2nd reuse	84.5 ± 1.7	97.1 ± 0.8	98.64 ± 0.31	95.21 ± 2.35
3rd reuse	82.6 ± 1.9	96.3 ± 0.9	97.82 ± 0.42	94.36 ± 2.48

## Data Availability

The original contributions presented in this study are included in the article. Further inquiries can be directed to the corresponding author.
